# Smallest Chimeras Under Repulsive Interactions

**DOI:** 10.3389/fnetp.2021.778597

**Published:** 2021-12-21

**Authors:** Suman Saha, Syamal Kumar Dana

**Affiliations:** ^1^ National Brain Research Centre, Gurugram, India; ^2^ National Institute of Technology, Durgapur, India; ^3^ Division of Dynamics, Lodz University of Technology, Lodz, Poland

**Keywords:** chimera, Josephson junction, repulsive coupling, ring of oscillators, libration motion, rotational motion

## Abstract

We present an exemplary system of three identical oscillators in a ring interacting repulsively to show up chimera patterns. The dynamics of individual oscillators is governed by the superconducting Josephson junction. Surprisingly, the repulsive interactions can only establish a symmetry of complete synchrony in the ring, which is broken with increasing repulsive interactions when the junctions pass through serials of asynchronous states (periodic and chaotic) but finally emerge into chimera states. The chimera pattern first appears in chaotic rotational motion of the three junctions when two junctions evolve coherently, while the third junction is incoherent. For larger repulsive coupling, the junctions evolve into another chimera pattern in a periodic state when two junctions remain coherent in rotational motion and one junction transits to incoherent librational motion. This chimera pattern is sensitive to initial conditions in the sense that the chimera state flips to another pattern when two junctions switch to coherent librational motion and the third junction remains in rotational motion, but incoherent. The chimera patterns are detected by using partial and global error functions of the junctions, while the librational and rotational motions are identified by a libration index. All the collective states, complete synchrony, desynchronization, and two chimera patterns are delineated in a parameter plane of the ring of junctions, where the boundaries of complete synchrony are demarcated by using the master stability function.

## 1 Introduction

Chimera states (Abrams and Strogatz, 2004; Sethia et al., 2008; Laing, 2009; Hagerstrom et al., 2012; Martens et al., 2013; Omelchenko et al., 2013; Gopal et al., 2014; Schöll, 2016; Hart et al., 2019; Majhi et al., 2019; Parastesh et al., 2020; Wang and Liu, 2020) became a paradigm of collective phenomena in dynamical systems that started with the first report by Kuramoto and Battogtokh (2002) of two coexisting synchronous and asynchronous groups of phase oscillators arranged in a ring and coupled in a non-local fashion. The main question was how the symmetry in a completely synchronous ensemble of identical oscillators breaks into two clusters, one synchronous group and another asynchronous group. How stable is this chimera state? In the beginning, it was apprehended that the chimera state is a transient behavior; the transient time increases with the size of a network (Wolfrum and Omel’chenko, 2011; Rosin et al., 2014). Later, it has been established that chimera states are possible stable states (Pecora et al., 2014; Omel’chenko, 2018; Laing, 2019) in an ensemble of identical oscillators and with symmetry in the connectivity matrix or the topology of a network. By this time, this phenomenon has been widely explored in single-layer networks (Abrams and Strogatz, 2004; Sethia et al., 2008; Laing, 2009; Hagerstrom et al., 2012; Martens et al., 2013; Omelchenko et al., 2013; Gopal et al., 2014; Hart et al., 2019; Majhi et al., 2019; Parastesh et al., 2020; Wang and Liu, 2020), multilayer networks (Ghosh and Jalan, 2016; Maksimenko et al., 2016; Sawicki et al., 2018; Ruzzene et al., 2020), and 3D networks (Maistrenko et al., 2015; Kasimatis et al., 2018; Kundu et al., 2019) with different forms of chimeras such as traveling chimera (Bera et al., 2016a; Omel’chenko, 2019; Dudkowski et al., 2019; Alvarez-Socorro et al., 2021) and spiral chimera (Martens et al., 2010; Gu et al., 2013). Various dynamical models (Hizanidis et al., 2015; Banerjee et al., 2016; Bera et al., 2016b; Saha et al., 2019) with different coupling schemes (Meena et al., 2016; Bera et al., 2017) and global coupling (Sethia and Sen, 2014; Yeldesbay et al., 2014; Hens et al., 2015a; Mishra et al., 2015) have been used for observing chimera patterns. The concepts of amplitude chimera (Hens et al., 2015a; Anna et al., 2016; Banerjee et al., 2018) and amplitude death chimera (Zakharova et al., 2014; Banerjee, 2015; Dutta and Banerjee, 2015) have been introduced. All the examples of chimera states revolve around the symmetry breaking of a complete coherent state into coherent and incoherent groups. This perception has been extended further to the observation of two coexisting subgroups of in-phase and antiphase oscillators (Maistrenko et al., 2017), which was also referred to as a chimera state. The necessary requirement of a large set of oscillators for chimera states to observe has also been relaxed with a smaller size of the network: Chimera patterns emerge in a set of four oscillators (Hart et al., 2016; Meena et al., 2016; Senthilkumar and Chandrasekar, 2019) and even three oscillators (Wojewoda et al., 2016; Maistrenko et al., 2017). In a laser system, delay in coupling has been used (Hart et al., 2016) for the observation of chimera states in four oscillators. A modified Kuramoto phase oscillators with inertia (Maistrenko et al., 2017) were used to demonstrate both in-phase and antiphase chimeras in three oscillators and later confirmed in experiments with three attractively coupled pendula (Wojewoda et al., 2016). In almost all the reported studies, attractive coupling has been used for the observation of chimeras, while a few examples are found to use a combination of both attractive and repulsive coupling (Hens et al., 2015a; Mishra et al., 2015) to originate chimera states in globally coupled oscillators.

We focus here on the role of repulsive interactions in the origin of chimera patterns in a small ensemble of three identical dynamical units. As reported earlier (Mishra et al., 2017; Ray et al., 2020), chimera states may emerge in a large ensemble of globally coupled Josephson junctions under repulsive interactions. Indeed, the repulsive coupling can induce chimera states in a smallest set of three Josephson junctions arranged in a ring. Interestingly, a single Josephson junction shows typical neuron-like spiking and bursting behaviors (Hens et al., 2015b; Hongray et al., 2015; Dana et al., 2006; Mishra et al., 2021), which is one of the reasons that encourage us to investigate the role of repulsive interactions (inhibitory in the sense of neuronal interaction) in a ring of three junctions. A single Josephson junction is represented by a resistance–capacitance shunted junction (RCSJ) circuit (Dana et al., 2001; Dana et al., 2006; Mishra et al., 2021), which remains in an excitable state until an external bias current is applied across the junction. The junction shows spiking limit cycle oscillation for bias current above a critical value. The dynamics of the junction also depends upon a damping-like parameter that is related to the shunted resistance and capacitance of the junction. It shows a bistable region, in the parameter space, where the limit cycle coexists with a steady state for a range of low damping. The RCSJ model can be represented by a second-order phase dynamics of the junction that governs the voltage drop across it. The RCSJ model also represents the dynamics of the simple pendulum model and discrete sine-Gordon equation. The dynamics of the junction is rotational when the trajectory of the junction makes a complete rotation around a cylindrical surface like an inverted pendulum, thereby originating a large-amplitude spiking oscillation. On the other hand, the junction may show, for an appropriate choice of parameters, librational motion when the trajectory of the junction dynamics is restricted to a small region of the cylindrical phase space. This appears like the small-amplitude oscillation of a pendulum.

Three Josephson junctions with identical parameters and by our choice of parameters are kept in rotational motion in an uncoupled state but kept away from the bistable region to avoid further complexity. The ring of three identical oscillators represents an all-to-all (or globally) coupled network and hence perfect symmetry. The junctions in the ring show 2*π*/3 out-of-phase motion in a cyclic order in time but emerge into a state of complete synchrony (CS) for repulsive interactions above a critical value. When the repulsive interactions in the ring are increased further, the symmetry is broken with the emergence of a sequence of desynchronous states, followed by chimera patterns: 1) two junctions are in complete synchrony (CS) in a state of rotational motion and one junction in incoherent rotational motion when they are all in a chaotic state, 2) two junctions in CS in rotational motion and one junction in incoherent libration when the dynamics of the junctions is periodic; this chimera pattern flips to two coherent junctions in libration and one in incoherent rotation for a change in initial conditions. We numerically delineate the different collective states in a two-parameter plane of the junctions using coherence measures and a libration index and use the master stability function (MSF) to demarcate the stable region of CS in the parameter plane.

## 2 Three Josephson Junctions in a Ring

**FIGURE 1:**
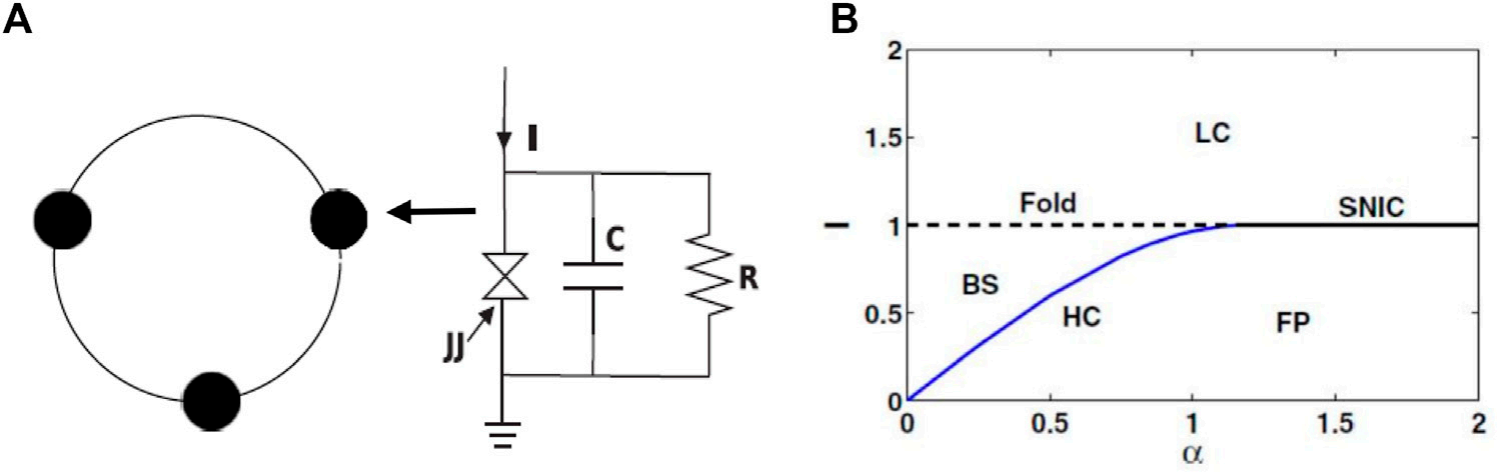
**(A)** Ring of three Josephson junctions (JJ). A schematic diagram of one RCSJ circuit shown at the right that represents the dynamics of each node in black circle. **(B)** Phase diagram of one RCSJ dynamics in an *α* − *I* parameter plane. Normalized critical current *I*_*c*_ = 1.0. The junction shows limit cycle (LC) oscillation for *I* > *I*_*c*_ = 1.0. The junction has a bistable (BS) region bounded by a fold bifurcation line (horizontal dashed line) and a homoclinic (HC) bifurcation curve (blue line). A stable fixed point (FP) coexists with a saddle for *I* < 1.0 in a region between the HC (blue line) and the saddle-node-invariant circle (SNIC) line. The junction transits to an LC state via SNIC bifurcation for *I* > 1.0.

A schematic diagram of the ring of three junctions is shown in Figure 1A. The RCSJ circuit is shown at right that represents the dynamics of each node. The dynamics of the ring of junctions is represented by the phase dynamics *θ*_*i*_ and voltage *v*_*i*_ (*i* = 1, 2, 3) across the *i*th junction given as follows (Josephson, 1962; Mishra et al., 2017):

$$\begin{array}{ccc} \dot{\theta_{i}} & = & v_{i}, \end{array}$$ (1)

$$\begin{array}{ccc} \dot{v_{i}} & = & I-\sin\theta_{i}-\alpha v_{i}+\varepsilon \left(v_{i-1}-2v_{i}+v_{i+1}\right), \end{array}$$

where *v*_*i*+1_ = *v*_1_, if (*i* + 1) > *n* and *v*_*i*−1_ = *v*_3_, if (*i* − 1) < 1. The initial conditions are randomly chosen from the range *θ*_*i*_(0) ∈ [ − *π*, *π*] and *v*_*i*_(0) ∈ [0.1, 1]. The damping parameter $\alpha ={\left[h/2\pi eI^{2}RC\right]}^{1/2}$ , where *h* is Planck’s constant and *e* is the electronic charge, and *R* and *C* are the resistance and capacitance of a junction, respectively. *I* is the external bias current normalized by the critical current *I*_*c*_ = 1.0 at each junction. The interaction between the nodes is established through junction voltages with a coupling strength *ɛ*, which is considered repulsive (negative) to observe our targeted chimera patterns. Figure 1B shows a phase diagram in an *α* − *I* plane for a single junction. The single junction remains excitable (FP) for bias current *I* < *I*_*c*_ and transits to limit cycle oscillation via saddle-node-invariant circle (SNIC) bifurcation for *I* > *I*_*c*_ and *α* > 1.19. In a lower range of *α* < 1.19 and *I* < *I*_*c*_, the junction shows bistability (BS) when the limit cycle coexists with a steady state. The bistable state transits to limit cycle oscillation via fold bifurcation when *I* > 1.0. The bistable region is bounded by fold bifurcation (dashed horizontal line) and homoclinic (HC) bifurcation (blue curve) in the *α* − *I* parameter plane. For our observation of chimeras, we make a choice of parameters *I* > *I*_*c*_ and *α* > 1.19 so as to obtain limit cycle oscillation (rotational motion) in all three junctions in uncoupled state and keep them away from the bistable region to avoid further complexity in dynamics.

## 3 Coherence and Libration Index

To check the CS state, we first calculate the error functions between all the pairs of nodes, separately, as follows:

$$e_{12}=\sqrt{\left\langle {\left(v_{1}-v_{2}\right)}^{2}\right\rangle };\;\;e_{13}=\sqrt{\left\langle {\left(v_{1}-v_{3}\right)}^{3}\right\rangle };\;\;e_{23}=\sqrt{\left\langle {\left(v_{2}-v_{3}\right)}^{2}\right\rangle },$$ (2)

where $\left\langle .\right\rangle$ indicates time average. We check all the three error functions simultaneously and detect a chimera state when two of the partial error functions are zero (say, *e*_12_ = 0 and *e*_13_ = 0).

The global error variable is then calculated as follows:

$$err=\left(e_{12}+e_{13}+e_{23}\right)/3.$$ (3)

For CS in the network, *err* = 0 and *err* ≠ 0 indicate partial synchrony or incoherence. We use a libration index (Mishra et al., 2017) to distinguish librational and rotational motion of the junctions in different collective states:

$$LI=\frac{1}{3}\overset{3}{\underset{j=1}{\sum }}\Theta_{j},$$ (4)

where Θ_*j*_ = Θ(*δ* − *m*_*j*_). Θ(.) is the Heaviside step function, where *δ* is an arbitrarily chosen small threshold and $m_{j}=1-0.5*\left(\max\left(\cos\left[\theta_{j}\left(t\right)\right]\right)-\min\left(\cos\left[\theta_{j}\left(t\right)\right]\right)\right)$ . The libration index becomes *LI* = 0 for oscillators in libration and *LI* = 1 when they are in rotational motion. A value of 0 < *LI* < 1 indicates the coexistence of librational and rotational motions in the ring of junctions.

**FIGURE 2:**
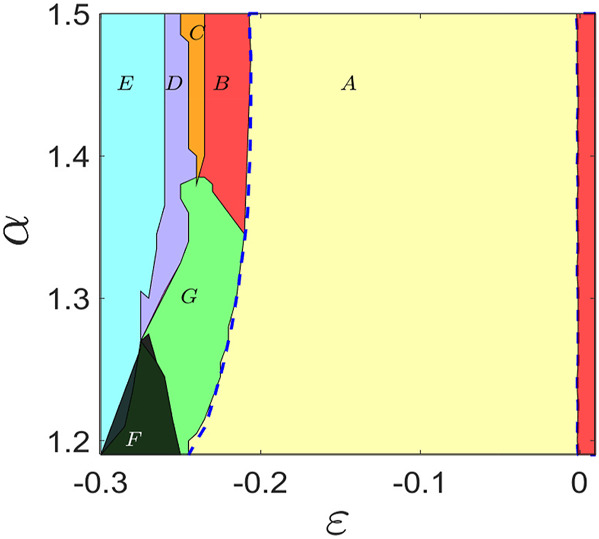
Phase diagram in an *α* − *ɛ* plane of a ring of three identical Josephson junctions. A large region of CS (region A, yellow) is demarcated within the boundaries of blue dashed lines, where *λ*_max_ is negative as estimated by using the MSF (8) for a range of repulsive coupling and *α* values. For larger repulsive coupling (beyond the blue dashed curve), CS becomes unstable, and there first emerges a state of asynchronous limit cycle dynamics (B, red), then asynchronous chaos (C, orange), followed by a reasonable range of rotational chimera (D, purple) in a chaotic state, and finally, a chimera state of mixed rotational and libration (E, cyan) in periodic motion for larger repulsive coupling and a broad range of *α* > 1.2. Stability of CS is also lost for attractive coupling (*ɛ* > 0, red region) as marked by a vertical dashed blue line. There are regions of mixed states F (black) and G (green) of coexisting chimera and desynchronization. Parameter *I* = 1.5.

For demarcation of the stable CS state in the parameter plane of the junctions in Figure 2, we numerically estimate the MSF (Pecora and Carroll, 1998) of identical junctions by using the variational equation of the ring of junctions. The dynamics of the *i*th node is ${\dot{x}}_{i}=F\left(x_{i}\right)+\varepsilon \sum_{j}G_{ij}H\left(x_{j}\right)$ . Now, let **x** = (**x**_1_, **x**_2_, …, **x**_*n*_), **F(*X*)** = [**F**(**x**_**1**_), **F**(**x**_**2**_), …, **F**(**x**_**n**_)], **H(*X*)** = [**H**(**x**_**1**_), **H**(**x**_**2**_), …, **H**(**x**_**n**_)] is the coupling matrix and **G** be the adjacency matrix {*G*_*ij*_}, then

$$\dot{x}=F\left(x\right)+\varepsilon G⊗H\left(x\right),$$ (5)

where ⊗ denotes the direct product. The variational equation of Eq. 5 by assuming *ξ*_*i*_ as a small perturbation in the *i*th node when *ξ* = (*ξ*_1_, *ξ*_2_, …, *ξ*_*n*_), (*i* = 1, 2, ..*n*) is given as follows:

$$\dot{\xi }=\left[1_{n}⊗DF+\varepsilon G⊗DH\right]\xi ,$$ (6)

when **H** is the coupling matrix **E** = (0 0; 1 0), implying coupling in second variable and *D* **H** = **E**. By a block diagonalization of the variational equation, each block will be of the form as follows:

$$\dot{\xi_{k}}=\left[DF+\varepsilon \gamma_{k}DH\right]\xi_{k},$$ (7)

where *γ*_*k*_ is the eigenvalue of **G**, *k* = 0, 1, 2, …, *n* − 1. For *k* = 0, we have the variational equation for the synchronization manifold *γ*_0_ = 0. The Jacobian matrices *D* **F** and *D* **H** are the same for each block, since they are evaluated on the synchronized state. Thus, for each *k*, the form of each block [Eq. 7] is same with only the scalar multiplier *ɛγ*_*k*_ differing. This leads us to the following formulation of the master stability equation and the associated MSF: We calculate the maximum Lyapunov exponents *λ*_*max*_ for the generic variational equation as follows:

$$\dot{\zeta }=\left[DF+\left(\alpha +i\beta \right)DH\right]\zeta ,$$ (8)

as a function of *α* and *β*. This yields the *λ*_*max*_ as a point on the real axis since we check the real part of the exponent, and **G** has only the real eigenvalues in our case. The sign of *λ*_*max*_ will reveal the stability of the eigenmodes, and hence, we have the MSF, which is used for delineating the boundary (blue dashed lines) of the CS region (A, yellow) as shown in Figure 2, where *λ*_*max*_ becomes negative.

## 4 Phase Diagram: Ring of Josephson Junctions

We draw a phase diagram to delineate the regions of a variety of collective states including the chimera patterns for the three junctions in the ring in an *α* − *ɛ* parameter plane in Figure 2. All the junctions are in out-of-phase for attractive coupling (*ɛ* > 0) when the junction voltages evolve in 2*π*/3 cyclic motion however become completely synchronized (CS) in the periodic state for a range of repulsive coupling (*ɛ* < 0). The boundaries of *ɛ* for CS are demarcated at two ends (by two blue dashed lines), which are indicated by a transition of *λ*_*max*_ to a negative value as estimated by using the MSF and perfectly matching with the numerically simulated boundaries of CS (region A, yellow) in the phase diagram. CS breaks down for attractive coupling at one end (red region) and larger repulsive coupling as well at the other end (regions B and G). CS transits to asynchronous periodic motion (B, red) of the junctions and a mixed state (G, green) for larger repulsive coupling. The chimera pattern (partial synchrony) coexists with complete asynchronous oscillation in region G (chaotic or higher periodic rotational motion). The ring of junctions becomes chaotic in region C (orange) and remains completely asynchronous. They form a chaotic chimera pattern with two coherent and one incoherent junctions in region D when all the junctions maintain rotational motion. Finally, a periodic state re-emerges in region E (cyan) with a chimera pattern. Two junctions become coherent in rotational periodic motion that coexists with a single junction in incoherent periodic libration. These chimera patterns flip with a new pattern when two junctions become coherent in libration and one in incoherent rotation; this flipping occurs due to sensitivity in initial conditions. Region F represents mixed states of coexisting chimera patterns and asynchronous states.

## 5 Smallest Chimeras in Three Junctions

**FIGURE 3:**
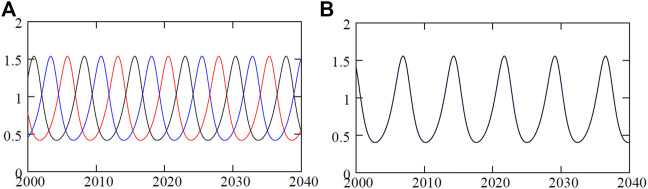
Time evolution of voltage in three junctions. Voltages *v*_*i*_ (*i* = 1, 2, 3) in three junctions evolve in **(A)** out-of-phase 2*π*/3 motion in a cyclic order in time (red, black, and blue lines) for *ɛ* = 0.02, **(B)** converge into complete synchrony of amplitude and phase (black lines) for *ɛ* = − 0.01. Parameters *α* = 1.45 and *i* = 1.5.

**FIGURE 4:**
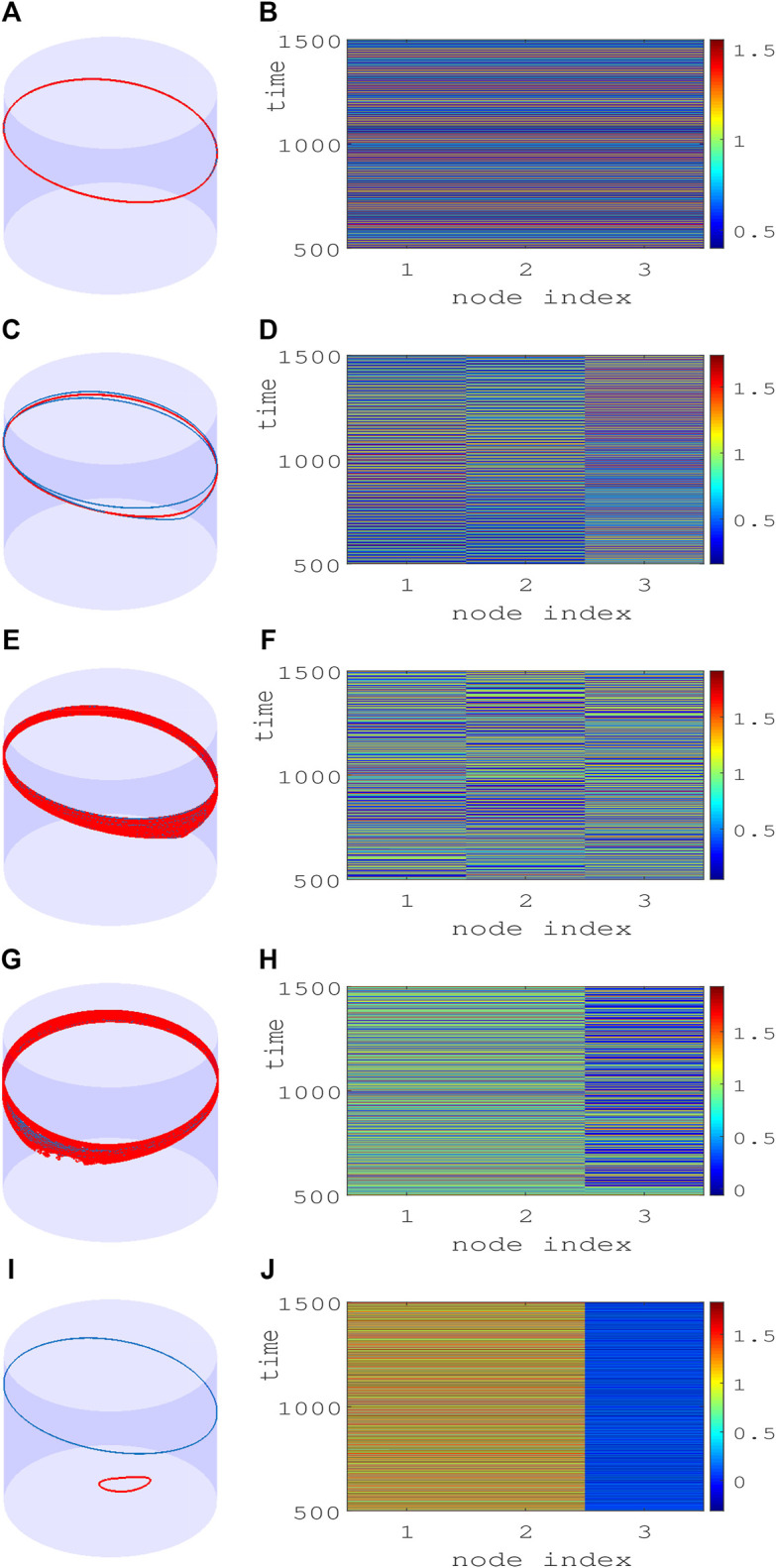
Smallest chimeras in a ring of three Josephson junctions **(A,C,E,G,I)**. Trajectory of junctions (panels in left column) in a *θ*-*v* cylindrical plane and **(B,D,F,H,J)** spatiotemporal evolution (panels in right column) of junction voltage *v* in three junctions. Region **(A)**: **(A)** One coherent periodic trajectory (red) is seen with **(B)** coherent evolution of all three junctions in time for *α* = 1.45, *ɛ* = − 0.02. Region **(B)**: Three distinctly separate periodic rotational trajectories **(C)** are seen in incoherent motion in time **(D)** for *α* = 1.45, *ɛ* = − 0.23. Region **(C)**: Three junctions are in chaotic rotational motion **(E)** with incoherent spatial evolution in time **(F)** for *α* = 1.45, *ɛ* = − 0.24. Region **(D)**: Chimera pattern in chaotic motion **(G)** when two junctions are evolving in coherent motion in time **(H)** and one is incoherent for *α* = 1.45, *ɛ* = − 0.25. Region **(E)**: Chimera pattern in dual motion. One coherent pair of junctions in rotational motion **(I)** and one junction in incoherent librational motion for *α* = 1.45, *ɛ* = − 0.265. Regions **(A–E)** are identified here as depicted in Figure 2. Color bars depict the amplitude limit of junction voltages.

Three identical junctions in the ring evolve out of phase in time in a cyclic order of 2*π*/3 phase lag for attractive coupling (*ϵ* > 0), and they evolve into a state of complete identical phase and amplitude for a repulsive coupling (*ϵ* < 0) above a threshold as decided by the MSF. The time evolution of the three junctions is plotted in Figures 3A,B when the junctions emerge in an out-of-phase state and CS state, for *ɛ* = 0.02 and *ɛ* = − 0.01, respectively. Both the out-of-phase and CS states are true for *N* > 3 number of junctions in the ring (results not shown here but checked for *N* = 4, 7, 8). For further illustration, the dynamics of three junction voltages *v* are demonstrated in Figure 4. We have already recognized two reasonably broad parameter regions of chimera patterns in the parameter planes (D and E). Now, we demonstrate their exemplary dynamics using the trajectories of the three junctions in a *θ* − *v* cylindrical plane and their corresponding spatiotemporal dynamics and how they evolve against varying repulsive coupling *ɛ* at a fixed *α* = 1.45. Figures 4A,B represent the CS state for repulsive interactions as confirmed by a plot of the trajectories (red) of three junctions in a *θ* − *v* cylindrical plane (left panel) and its corresponding spatiotemporal plot at the right panel. For stronger repulsive interaction, Figures 4C,D show that CS breaks down, although the junctions maintain rotational periodic motion; however, their trajectories are distinctly different (blue, red, and green) which is confirmed by their incoherent motion in the spatiotemporal plot (right panel). The junctions become chaotic for larger repulsive current yet continue with rotational incoherent motion as depicted by their trajectories and spatiotemporal evolution in Figures 4E,F. The first sign of the chimera pattern appears for further increase in repulsive coupling when all the junctions are in chaotic rotational motion, as shown in Figure 4G. However, two of the junctions (indices 1 and 2) are evolving in the coherence of amplitude and phase, while the third junction (index 3) is incoherent in rotational motion as depicted in the spatiotemporal plot in Figure 4H. The collective dynamics of three junctions then switches to periodic motion for a further increase in repulsive coupling. Interestingly, the junctions split into two groups when a pair of junctions remains in coherent rotation (blue), as shown in Figure 4I, and one junction 3) switches to librational motion (red) and incoherent to the other two junctions (1 and 2). This state of coexisting coherence and incoherence in two groups as typically defined as a chimera pattern is confirmed by their spatiotemporal evolution in Figure 4J.

## 6 Conclusion

The role of repulsive interactions in the origin of chimera patterns in a small set of three dynamical units in a ring has been explored here. A ring of three nodes with all-to-all coupling is quite often considered as a network motif (Milo et al., 2002). We used the exemplary model of the superconducting Josephson junction as dynamical units that basically represent a second-order phase dynamics under damping and external bias. Noteworthy that this model also represents the dynamics of a simple pendulum and the discrete sine-Gordon equation. For attractive coupling, three junctions maintain an out-of-phase in 2*π*/3 alternate rotational motion in a cyclic order, which is counterintuitive to the common notion of synchrony under attractive coupling. Interestingly, three junctions emerge into CS for repulsive coupling only. We are still working on this strange collective behavior but yet to find a complete explanation, although we make some inroads into why and when such a behavior may emerge that is to be reported in the future. However, we have checked this new paradigm of CS using the MSF that establishes its stability in a range of repulsive coupling independent of the number of junctions in the ring. Chimera patterns emerge in three junctions beyond the CS state when it breaks down for larger repulsive coupling. We define chimera states here as two coherent junctions and one junction incoherent to the other two that follow the standard definition of chimera states in the literature. We identified two different patterns of chimera states for repulsive interactions. Three junctions may remain in chaotic rotational motion, yet two of them evolve in coherence, while the third junction also evolves in rotational motion but incoherent to the other two junctions. In another kind of chimera pattern, for larger repulsive coupling, two junctions may evolve in coherent rotational motion, while the third junction switches to small-amplitude librational motion and becomes incoherent to the other two junctions. This chimera pattern is sensitive to initial conditions when the chimera pattern may flip to coherent oscillators in coherence, while the third is in incoherent rotational motion. We cannot designate the chimera patterns as strong chimera (Zhang and Motter, 2021). However, some recent reports consider this latter chimera pattern as a solitary state (Jaros et al., 2018; Semenova et al., 2018; Rybalova et al., 2021) since a single oscillator behaves differently, in the dynamical sense, from the other two coherent oscillators.

## Data Availability

The original contributions presented in the study are included in the article/Supplementary Files, and further inquiries can be directed to the corresponding author.
